# Climate heterogeneity shapes phylogeographic pattern of *Hippophae gyantsensis* (Elaeagnaceae) in the east Himalaya‐Hengduan Mountains

**DOI:** 10.1002/ece3.10182

**Published:** 2023-06-09

**Authors:** Ting Xu, Ruixue Wang, Qiong La, Takahiro Yonezawa, Xinyi Huang, Kun Sun, Zhiping Song, Yuguo Wang, Igor V. Bartish, Wenju Zhang, Shanmei Cheng

**Affiliations:** ^1^ Institute of Biodiversity Science, School of Life Sciences Fudan University Shanghai China; ^2^ College of Life Sciences Northwest Normal University Lanzhou China; ^3^ Department of Biology Tibet University Lhasa China; ^4^ Institute of Botany of the Czech Academy of Sciences Pruhonice Czech Republic; ^5^ Laboratory of Subtropical Biodiversity Jiangxi Agricultural University Nanchang China

**Keywords:** climatic heterogeneity, east Himalaya‐Hengduan Mountains, *Hippophae gyantsensis*, phylogeography, quaternary climatic fluctuations

## Abstract

The interaction of recent orographic uplift and climate heterogeneity acted as a key role in the East Himalaya‐Hengduan Mountains (EHHM) has been reported in many studies. However, how exactly the interaction promotes clade diversification remains poorly understood. In this study, we both used the chloroplast *trn*T‐*trn*F region and 11 nuclear microsatellite loci to investigate the phylogeographic structure and population dynamics of *Hippophae gyantsensis* and estimate what role geological barriers or ecological factors play in the spatial genetic structure. The results showed that this species had a strong east–west phylogeographic structure, with several mixed populations identified from microsatellite data in central location. The intraspecies divergence time was estimated to be about 3.59 Ma, corresponding well with the recent uplift of the Tibetan Plateau. Between the two lineages, there was significant climatic differentiation without geographic barriers. High consistency between lineage divergence, climatic heterogeneity, and Qingzang Movement demonstrated that climatic heterogeneity but not geographic isolation drives the divergence of *H. gyantsensis*, and the recent regional uplift of the QTP, as the Himalayas, creates heterogeneous climates by affecting the flow of the Indian monsoon. The east group of *H. gyantsensis* experienced population expansion *c*. 0.12 Ma, closely associated with the last interglacial interval. Subsequently, a genetic admixture event between east and west groups happened at 26.90 ka, a period corresponding to the warm inter‐glaciation again. These findings highlight the importance of the Quaternary climatic fluctuations in the recent evolutionary history of *H. gyantsensis*. Our study will improve the understanding of the history and mechanisms of biodiversity accumulation in the EHHM region.

## Graphical abstract

It is a heterogeneous climate but not a geographic barrier in the EHHM that shaped phylogeographic structure of *H. gyantsensis*. The role of high mountains in directing the evolution of *H. gyantsensis* seems largely to be in creation of highly heterogeneous climates by affecting the flow of the Indian monsoon in this region.

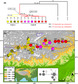

## 1 INTRODUCTION

The East Himalaya‐Hengduan Mountains (EHHM) is one of the most well‐known biodiversity hotspots in the world (Myers et al., 2000). This region is not only the diversification or/and origin center of many taxa (Hu, 1994; Jia et al., 2012; The Comprehensive Scientific Expedition to the Qinghai‐Xizang Plateau, 1986; Wu, 1988) but also has been considered a major glacial refuge during the glacial period for many species (Frenzel et al., 2003; Wu, 1988; Yang et al., 2008). Over the last two decades, this region has become recognized as an important issue in biodiversity and evolutionary research (Favre et al., 2015; Qiu et al., 2011; Renner et al., 2016; Spicer et al., 2021; Wang et al., 2011; Wen et al., 2014). The EHHM region has extremely complex topography and variable climate, and climatic gradients are steep from west to east (The Comprehensive Scientific Expedition to the Qinghai‐Xizang Plateau, 1997). Also, here orogenesis has been strong since the early Cenozoic (Deng & Ding, 2015; Ding & Zhong, 2013; Royden et al., 2008), which strongly impacted the stability of environments in this region. The complex topography and highly variable climate in the EHHM are the main factors behind highly heterogeneous habitats or various niches observed in the region, which have been considered to account for the extremely rich biodiversity here (Wu, 1988; Xing & Ree, 2017; Zhang et al., 2009).

Generally, high mountains and wide rivers have significant effects on divergence and phylogeographic structure of species by creating strong geographic barriers for seed dispersal (Avise, 2000). Indeed, these effects have been demonstrated on some species in the EHHM region (Liu et al., 2009; Meng et al., 2007; Wen et al., 2014; Xu et al., 2010). However, some recent studies also have indicated that ecological factors, especially climate, might also play an important role in driving cryptic speciation or intra‐specific divergence (Fan et al., 2013; Liu et al., 2013; Pinto‐Carrasco et al., 2021; Yang et al., 2012; Zhang et al., 2016). Nevertheless, as Favre et al. (2015) reviewed, despite a growing number of studies, the specific mechanisms behind origin and evolution of biodiversity hotspots associated with the Tibetan Plateau remain poorly understood and further studies are needed.

For the patterns of biodiversity and climate change on the Qinghai Tibetan Plateau (QTP), the most serious dilemma we face is that we still cannot confirm when and how the QTP (the Himalaya Mountains (HM), one of the main constituent terranes of QTP), reached its modern elevation. There has been considerable diversity of opinions about the process and date of the plateau uplift (Deng & Ding, 2015; Renner, 2016; Royden et al., 2008; Spicer et al., 2021; Wang et al., 2014). These opinions often yield conflict inferences owing to different lines of evidences. Some researchers hold that the central region of the plateau rose to its present height as early as 40 million years ago (Ma), with subsequent outward extensions by the early Miocene (Rowley & Currie, 2006), while some other scholars suggested that the high central plateau was not formed until the Neogene (Su et al., 2019). Meanwhile, some other studies indicated that the uplift of the plateau reached over 4000 m average elevation only by 15 Ma (Coleman & Hodges, 1995; Spicer et al., 2003) or even 8–10 Ma (Deng & Ding, 2015; Harrison et al., 1992). In recent decades, drastically different hypothesis for recent and rapid uplift of the QTP at about 3.6 Ma was also proposed (Cui et al., 1996; Li et al., 2013, 2015; Shi, 1998). Thus, heated debates about the evolution history of QTP uplift still continue, in spite of unceasing accumulation of evidence from tectonics, fossils, isotopes, and climate simulations. Hence, in the region, studies of phylogeographic and phylogenetic patterns, which can link organic diversification with geological history and environmental change, are still needed.

*Hippophaë* is a genus of Elaeagnaceae and it may be one of the most appropriate taxa to clarify the mentioned above relationships. The EHHM region is the diversification center of this genus, and six of seven species of the genus are distributed there (Jia & Bartish, 2018; Lian et al., 2006; Swenson & Bartish, 2002). *Hippophaë* has a long evolutionary history (from the Eocene or early Oligocene) as indicated by both paleobotanic (Akkiraz et al., 2011; Miao et al., 2013) and molecular data (Bartish, 2016; Jia & Bartish, 2018). The range and the age of the genus both imply that the geological and climatic changes of the EHHM region should have influenced its evolution. Our previous study on *H. tibetana* indicates a link between the uplift of QTP during the last 3~4 Ma, and that the climate change might play an important role in driving intraspecific diversification in *H. tibetana* (Wang et al., 2010). Another species of this genus, *H. gyantsensis* (Rousi) Lian, may provide even better opportunity to test the presented above hypothesis. This species mainly occurs in the valleys of the middle Yarlung Zangbo River (YZR), only grows on riverbanks and floodplains, and ranges from 3000 to 4600 m in altitude (Lian et al., 2006). The distribution range of *H. gyantsensis* is now limited to the central Himalaya, not EHHM region, but we found several putative hybrid populations of this species in the Hengduan Mountains (personal communication), so we still focused on the EHHM region in this study. Age of the species was estimated be to the Early Miocene (Jia et al., 2016; Jia & Bartish, 2018) and its origin may be explained by several ancient hybridization events within the genus (Jia et al., 2016). Comparing with *H. tibetana*, this species has a continuous distribution and covers various climate zones from west to east (The Comprehensive Scientific Expedition to the Qinghai‐Xizang Plateau, 1984). Importantly, there are no significant geographic barriers in its main distribution because its range is along the YZR valley, which is the channel for seed dispersal by birds. The fruits of *Hippophaë* plants are important food sources for many birds in the QTP (Lu et al., 2005) and thus can be dispersed over a long distance, which enables us to exclude the effects of strong geographic barriers and to test the effect of climate dynamics on species diversification.

Up to now, most phylogeographic studies in EHHM region concentrated on alpine plants, and very few of them studied the plants from the YZR there (Cheng et al., 2017; Wang et al., 2019). However, species growing in YZR are more sensitive to detecting the influence of climate dynamics. Here, through the inspection into the phylogeography of *H. gyantsensis* using both chloroplast DNA (cpDNA) and microsatellite fragments, we aimed to answer the following specific questions: (1) Does *H. gyantsensis* display significant phylogeographic structure? (2) Which role did climatic dynamics and the uplift of QTP play in shaping the phylogeographic structure of *H. gyantsensis*, respectively? (3) What are the historical factors related with the population demography?

## 2 MATERIALS AND METHODS

### 2.1 Population sampling, DNA sequencing, and microsatellite genotyping

**FIGURE 1:**
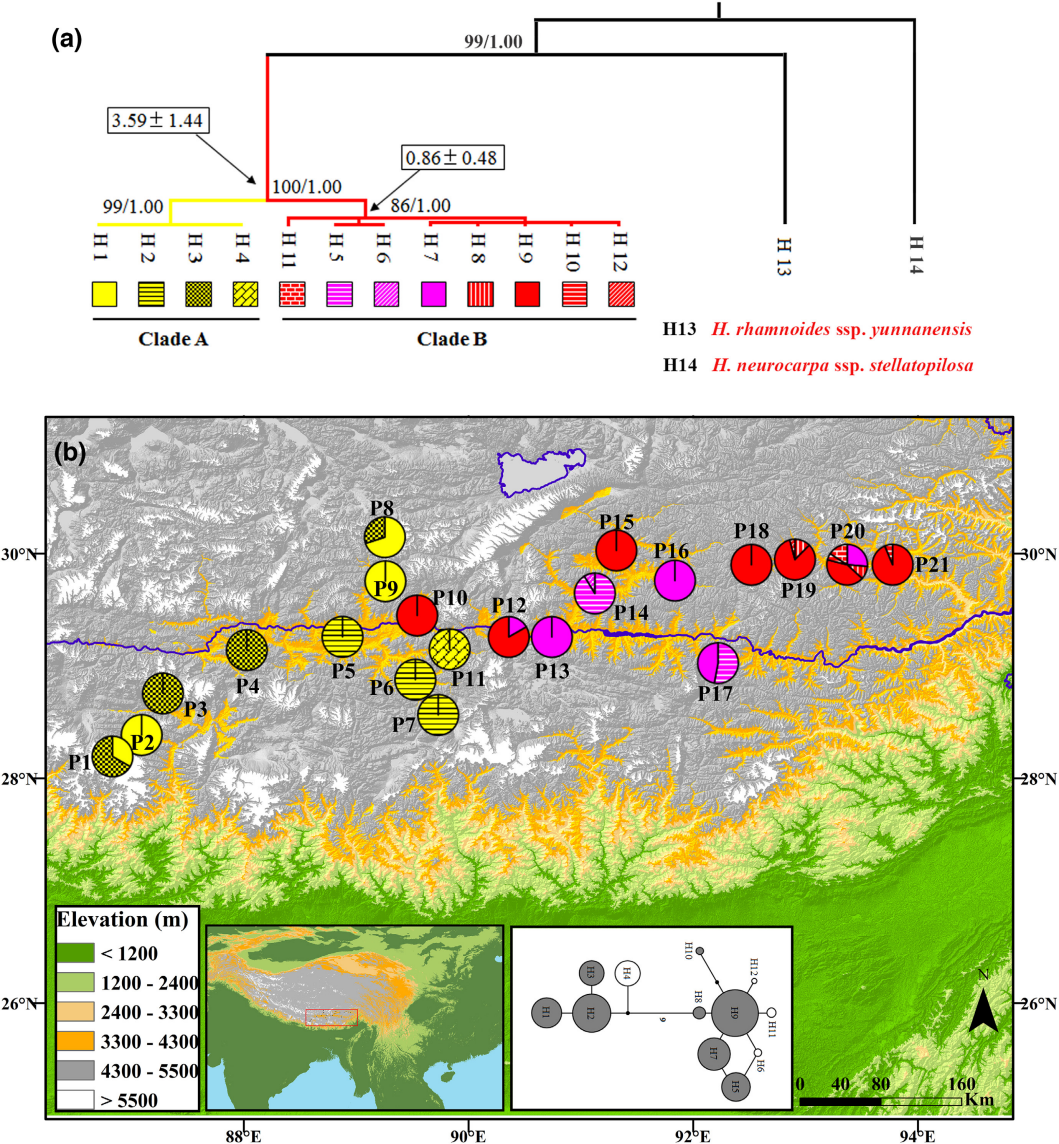
(a) Phylogenetic relationship of cpDNA haplotypes of *Hippophae gyantsensis* based on maximum likelihood (ML) method. Numbers near the branches are bootstrap values for ML analyses (left) and Bayesian posterior probabilities (right) from left to right; divergence time estimated in million years ago ± SE are given in the rectangular boxes. (b) Geographic locations of cpDNA haplotypes of *H. gyantsensis*. Pie charts show the proportions of cpDNA haplotypes within each population.

A total of 22 populations were sampled across the whole geographic range of *H. gyantsensis*, of which 21 (P1–P21) were *H. gyantsensis*, and one (P22) was *H. rhamnoides* subsp. *yunnanensis*. In each population, fresh leaves of 5–24 individual plants separated by at least 10 m were collected and dried by silica gel. The location and sample size of each population were shown in Figure 1 and reported in Table 1.

**TABLE 1:** Geographic locations and genetic diversity of 21 populations of *Hippophae gyantsensis* based on chloroplast *trn*T‐*trn*F region and 11 microsatellite loci, respectively.

Population	Location	Longitude (E)	Latitude (N)	Altitude (m)	Voucher	*N*	Haplotypes (%)	*H*_d_ cpDNA	*H*_e_ SSR
P1	Dingri	86°49.829′	28°18.398′	4600	LOZ‐024	6	H1 (33), H3 (67)	0.533	0.320
P2	Dingri	87°00.434′	28°24.994′	4173	HT0102	12	H1 (100)	0.000	0.381
P3	Dingri	87°11.253′	28°40.032′	4250	LOZ‐027	5	H3 (100)	0.000	0.329
P4	Lazi	88°02.497′	29°08.890′	4100	LOZ‐025	9	H3 (100)	0.000	0.286
P5	Rikaze	88°53.652′	29°17.243′	3850	LOZ‐002	23	H2 (100)	0.000	0.437
P6	Jiangzi	89°32.821′	28°57.479′	3993	LOZ‐003	24	H2 (100)	0.000	0.404
P7	Kangma	89°40.336′	28°37.116′	4150	LOZ‐004	5	H2 (100)	0.000	0.330
P8	Nanmulin	89°11.698′	30°07.209′	4550	ZLX11‐14	10	H1 (70), H3 (30)	0.467	0.251
P9	Nanmulin	89°13.940′	29°49.372′	4130	ZLX11‐15	10	H1 (100)	0.000	0.315
P10	Nanmulin	89°37.838′	29°24.600′	3800	LOZ‐001	11	H9 (100)	0.000	0.349
P11	Renbu	89°45.906′	29°17.249′	3828	HT0092	22	H4 (100)	0.000	0.348
P12	Nimu	90°24.395′	29°16.228′	3669	LZ07‐1	12	H7 (17), H9 (83)	0.303	0.209
P13	Qushui	90°41.121′	29°18.925′	3570	HT0094	12	H7 (100)	0.000	0.201
P14	Lahsa	91°06.611′	29°41.032′	3650	LOZ‐028	23	H5 (91), H6 (9)	0.166	0.258
P15	Linzhou	91°16.046′	29°58.594′	3876	GZ‐01	10	H9 (100)	0.000	0.265
P16	Mozhugongka	91°56.456′	29°45.260′	4000	LZ001	9	H7 (100)	0.000	0.334
P17	Qusong	92°10.887′	29°04.333′	3866	HT0106	17	H5 (53), H7 (47)	0.529	0.273
P18	Gongbujiangda	92°38.190′	29°52.356′	4039	HT0112	19	H9 (100)	0.000	0.366
P19	Gongbujiangda	92°57.583′	30°01.482′	3639	HT0111	24	H8 (13), H9 (83), H12 (4)	0.301	0.227
P20	Gongbujiangda	93°23.300′	29°53.643′	3353	HT0110	19	H7 (26), H8 (11), H9 (42), H10 (5), H11 (16)	0.754	0.209
P21	Gongbujiangda	93°41.490′	29°50.879′	3188	HT0109	16	H9 (94), H10 (6)	0.125	0.283
Mean						298		0.827	0.304
P22	Milin	94°03.857′	29°11.710′	2866	HT0107	21	H13 (100)		

Abbreviations: *H*_d_, haplotype diversity; *H*_e_, expected heterozygosity; *N*, sample size.

Total genome DNA was extracted using a modified CTAB method (Doyle & Doyle, 1987). The cpDNA region *trn*T*‐trn*F was amplified using primers “a” and “MR”, “c” and “f”, of which “MR” (5′ TAACGCAACGCAGCCAAC 3′) was designed in this study and others were from Taberlet et al. (1991). Reagents used and PCR conditions followed the protocol described by Wang et al. (2010). Sequencing reactions were performed with PCR primers “a”, “MR”, and “f”, using BigDye^®^ Terminator v3.1 Cycle Sequencing Kits and 3730*xl* DNA Analyzer (Applied Biosystems™).

Here, 11 polymorphic microsatellite markers were also developed from transcriptome of *H. gyantsensis* (Tang et al., personal communication, 2023). Primer sequences and amplification conditions were as described in Table S1. Forward primers were 5′‐end labeled using M13 (5′‐CACGACGTTGTAAAACGAC‐3′). The 10 μL PCR mix contained 6.15 μL dd H_2_O, 1 μL 10 PCR Buffer (Mg^2+^ free), 0.6 μL Mg^2+^ (25 mM), 0.8 μL dNTP (2.5 mM), 0.04 μL MF‐primer (10 μM), 0.36 μL M13 primer (10 μM), 0.4 μL R‐primer (10 μM), 0.15 μL Taq polymerase (5 U/μL) and 0.5 μL template DNA. M13 primers were 5′‐end fluorescently labeled by FAM, ROX, JOE, respectively. We performed PCRs with the following profile: initial denaturing of 5 min at 94°C, followed by 30 cycles of 94°C for 30 s, 50–55°C for 30 s, 72°C for 30 s, and a final step at 72°C for 10 min. The PCR products were visualized on 1% TAE agarose gels and then were run on an ABI 3730 XL DNA Sequencer. Finally, we genotyped individuals with GeneMapper v3.7 (Applied Biosystems).

### 2.2 Chloroplast data analysis

The cpDNA *trn*T*‐trn*F sequences were aligned with CLUSTAL X (Thompson et al., 1994), corrected manually in MEGA version 5 (Tamura et al., 2011), and then assigned to different haplotypes using DnaSP version 6 (Rozas et al., 2017). Sequences of each haplotype have been deposited to GenBank (KJ542834–KJ542846, KJ542860).

Phylogeny of all cpDNA haplotypes of *H. gyantsensis* was reconstructed with outgroups consisting of *H. rhamnoides* subsp. *yunnanensis* and *H. neurocarpa* ssp. *stellatopilosa*, as suggested by topology of *Hippophae* tree from Jia and Bartish (2018). Simultaneously, we downloaded all the public *trn*L‐*trn*F and ITS sequences (31 and 22, respectively) of *H. gyantsensis* from GenBank, of which 8 and 14 were respectively used in Jia et al. (2016). We used all these sequences for reconstructing the phylogenetic relationships within *H. gyantsensis* based on both *trn*L‐*trn*F and ITS fragments. Phylogeny reconstruction with Maximum likelihood (ML) method was carried out using RAxML‐VI‐HPC (Stamatakis, 2006). Clade support was evaluated by bootstrap (1000 replicates). Bayesian Inference was conducted with BEAST version 1.7.4 (Drummond et al., 2012), employing the same model as used in ML analysis. The condition of Markov chain Monte Carlo (MCMC) was as follows: the total generation length was 10,000,000 generations, and trees were sampled each 1000 generations. The first 1,000,000 generations were discarded as burn‐in. Convergences of each parameter were confirmed by the TRACER ver. 1.5, and ESS (effective sample size) of all parameters were larger than 200. The Network of cpDNA haplotypes was reconstructed with Network 10.3 (available at https://www.fluxus‐engineering.com/sharenet.htm) using the Median‐joining method (Bandelt et al., 1999) and MP calculation (Polzin & Daneshmand, 2003).

Haplotype diversity (*H*_d_) and nucleotide diversity (*π*) of all the populations were estimated by DnaSP version 6. Average diversity within populations (*H*_S_), total gene diversity (*H*_T_), and two coefficients of population differentiation (*G*_ST_ and *N*_ST_) were calculated using PERMUT 1.0 (Pons & Petit, 1996). *G*_ST_ (coefficient of genetic variation over all populations) is only based on allele frequencies while *N*_ST_ takes the similarities of alleles into account, therefore significantly larger *N*_ST_ than *G*_ST_ suggests that similar alleles tend to be geographically closer, indicating significant phylogeographic structure. The significance was tested by 1000 permutations.

The demographic history of *H. gyantsensis* was inferred by pairwise mismatch distribution (Rogers & Harpending, 1992; Slatkin & Hudson, 1991) in Arlequin 3.5 (Excoffier & Lischer, 2010). When the null hypothesis was not rejected, the time since expansion (*t*) was estimated according to the formula *t* = *τ*/2*u* (Rogers & Harpending, 1992), the value *u* = *μkg*, where *μ* is the substitution rate, *k* is the average sequence length, and *g* is the generation time. Here, it was approximately estimated to be 5 years for *H. gyantsensis* (Bartish et al., 2006). In addition, neutral tests with Tajima's *D* (Tajima, 1989) and Fu's *F*_S_ (Fu, 1997) were also conducted.

To define groups of populations, space analysis of molecular variance (SAMOVA) for cpDNA was conducted with SAMOVA 1.0 (Dupanloup et al., 2002). In this analysis, we set the number of groups (*K* value) from 2 to 6. According to the definition of groups by SAMOVA, an analysis of molecular variance (AMOVA) was also conducted using Arlequin 3.5 to calculate the molecular variance. The significance tests were based on 1000 permutations.

We also tested whether the molecular clock was hold or not by using the BASEML program of PAML version 4 (Yang, 2007) on the basis of the ML tree topology. When the molecular clock hypothesis was not rejected, divergence times were estimated by the molecular clock. The substitution rate for the *trn*L*‐trn*F region in *Phylica* (Rhamnaceae), 4.87 × 10^−10^ substitutions per site per year (s/s/y), was adopted, which was summarized by Richardson et al. (2001) and used for *H. tibetana* (Wang et al., 2010). We used this calibration in our dating analyses. Jia and Bartish (2018) dated chloroplast phylogenies of Elaeagnaceae and *Hippophaë* by first resolving phylogenetic relationships within these taxa using a concatenated database of five chloroplast loci. They then calibrated these phylogenies by a fossil record of *Shepherdia* in North America from the Late Eocene. According to these authors, mean age of the stem node of *H. gyantsensis* is about 20 Ma. We refrained from using this calibration because our interest was mainly in intraspecific differentiations within *H. gyantsensis*, which were found by Jia and Bartish (2018) to be much younger evolutionary events.

### 2.3 Microsatellite data analysis

**FIGURE 2:**
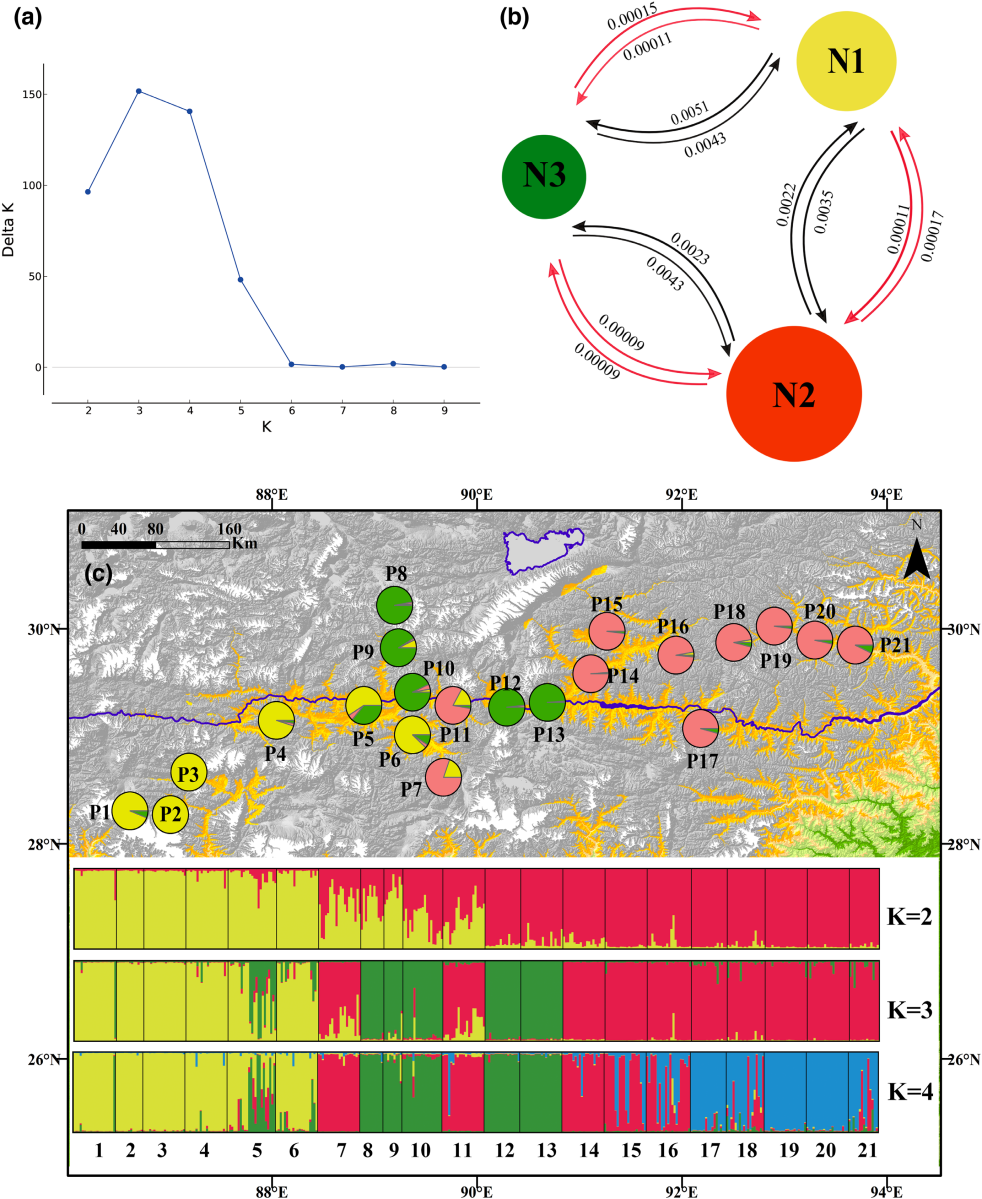
Bayesian inference analyses of 11 nuclear microsatellite loci for *Hippophae gyantsensis*. (a) Distribution of Delta *K*; (b) Estimates of historical (red lines) and contemporary (black lines) gene flow among three genetic clusters; (c) Geographic distribution of three genetic clusters (optimal number) and structure results of *K* = 2–4 revealed by STRUCTURE analysis.

To measure the level of genetic diversity and genetic differentiation for microsatellite data, several programs were performed as described below. First, the software MicroChecker v2.2.3 (Van Oosterhout et al., 2004) was used to estimate null alleles and correct the data. Second, tests for Hardy–Weinberg equilibrium and polymorphic information content (PIC) were calculated using Cervus 3.0.7 (Kalinowski et al., 2007). Third, several parameters of genetic population, including number of alleles per locus (*N*_a_), Shannon's Information index (*I*), observed heterozygosity (*H*_o_), expected heterozygosity (*H*_e_), genetic differentiation coefficient (*F*_st_), and fixation Index (*F*) were estimated using GenAlEx v6.5 (Peakall & Smouse, 2012). In addition, we assessed population genetic structure under admixture model using the Bayesian method implemented in Structure 2.3.4 (Hubisz et al., 2009). The number of clusters (*K*) ranged from one to ten using 20 independent runs for each value of *K*. Each run comprised a burn‐in of 10^6^ generations, and followed by 10^6^ MCMC steps. The optimal *K* was determined by log‐likelihood value and Δ*K* statistics (Evanno et al., 2005). The population clusters were visualized using the software Distruct 1.1 (Figure 2; Rosenberg, 2004). Subsequently, AMOVA was performed to partition total genetic variation within and among populations based on the result of structure analysis with 1000 permutations using Arlequin 3.5.

**FIGURE 3:**
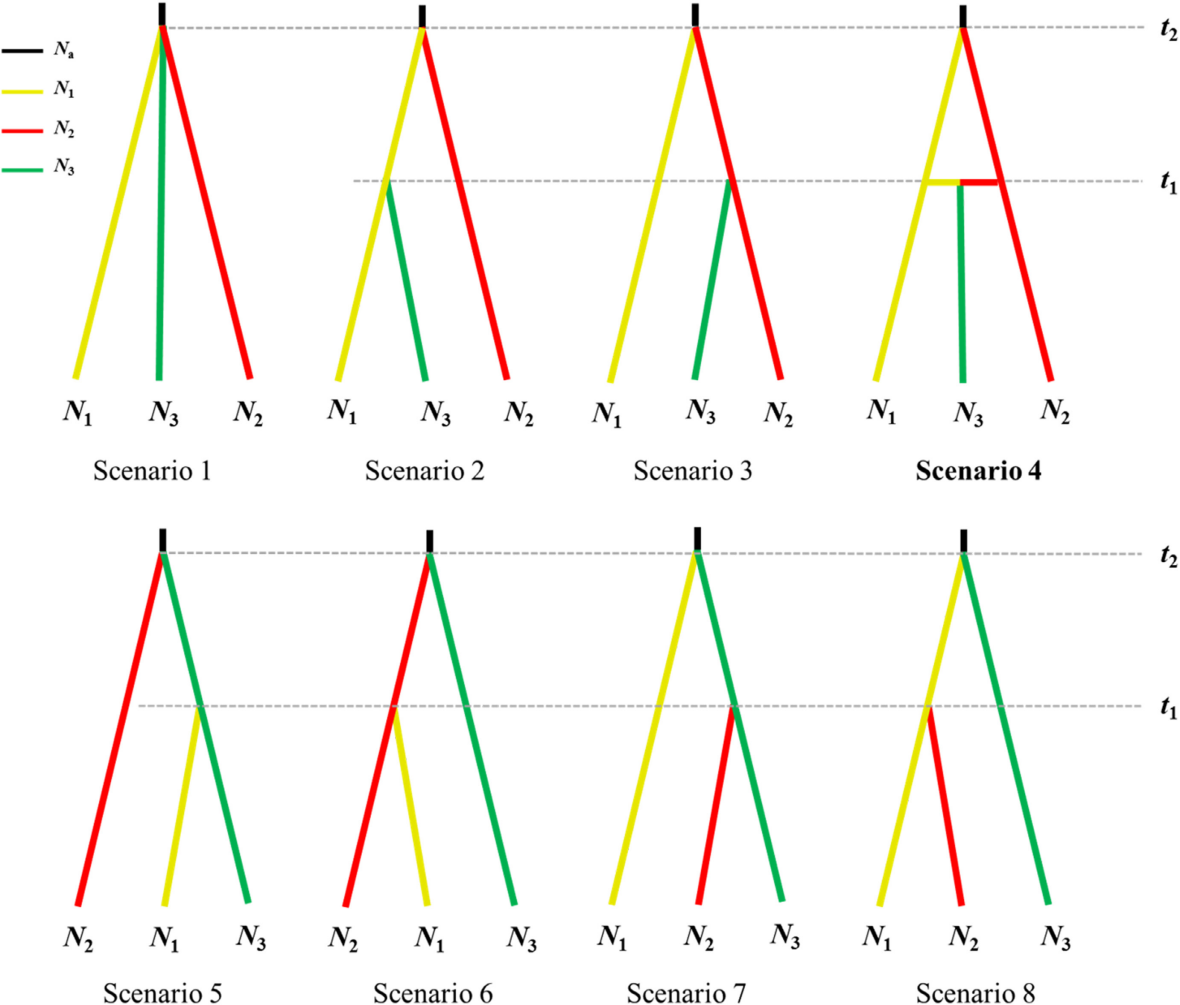
Alternative phylogeographic scenarios of three groups of *H. gyantsensis* evaluated by approximate Bayesian computation. *N*_1_, western cluster; *N*_2_, eastern cluster; *N*_3_, central cluster (i.e. mixed populations).

In addition, we evaluated the statistical support for eight alternative phylogeographic scenarios of the divergence history of *H. gyantsensis* (Figure 3) based on the above‐mentioned structure analysis using an Approximate Bayesian Computational (ABC) approach. We did not use the chloroplast data for ABC modeling due to insufficient variation in the sampled DNA fragment. Besides, unlike microsatellites, cpDNA represents only a small and specific part of the total genome of the species. Three groups were seta as western cluster (*N*_1_), eastern cluster (*N*_2_), and central cluster (*N*_3_) after removing three mixed populations (i.e. P5, P7, and P11). The eight scenarios were designed in detail as below: in the first scenario, *N*_1_, *N*_2,_ and *N*_3_ derived from an ancestral population at the same time (*t*_2_). The other scenarios showed some (presumably most likely) of the possible patterns of divergence events among the three groups, of which the fourth scenario showed that *N*_3_ was formed from a genetic admixture event between *N*_1_ and *N*_2_ at time *t*_1_. We gave a uniform prior probability and ran 8 × 10^6^ simulations under each scenario using DIYABC 2.1.0 (Cornuet et al., 2014), of which 10% was used to estimate the relative posterior probability with 95% credible intervals via logistic regression and posterior parameter distribution (Appendix Figure S1; Table S2). We chose the most likely scenario according to the assessment of the posterior probability. The divergence times were calculated in generations and were finally converted into years by multiplying the number of generations by generation time.

Finally, Migrate 3.0 (Beerli, 2008) and BayesAss 3.0 (Wilson & Rannala, 2003) were used to compare migration rates over historical and contemporary timescales, respectively. Migrate uses the coalescent method to estimate the gene flow *M* (*m*/*μ*) between pairs of clusters over a long period (ca. 4Ne generations in the past). In contrast, BayesAss uses the Bayesian approach and MCMC sampling to generate migration rate of the last few generations (Wilson & Rannala, 2003). We compared the values of m directly estimated by BayesAss and m (*M* × *μ*) generated by Migrate by dividing all *M* values by an estimated mutation rate of 2.92 × 10^−5^ from ABC simulation.

### 2.4 Climatic data analysis

To identify the climatic factors potentially associated with the divergence between groups of *H. gyantsensis*, we compared recent (c. 1950–2000) data of 19 BIOCLIM variables (Hijmans et al., 2005) between the groups. We extracted temperature and precipitation values from the BIOCLIM data sets with a grid size of 30″ (*c*. 1 km^2^ at the equator) using ArcGIS version 10.2 (ESRI Inc.) according to the GPS geographical coordinates of each population. For testing ecological differentiation between groups of populations from different parts of the range, we compared the average of each variable between groups of populations by two‐tailed *t*‐tests and evaluated the intergroup variance by one‐way analysis of variance (ANOVA), using R version 3.0.0. Finally, the significance level of each variable was corrected by Bonferroni method.

## 3 RESULTS

### 3.1 Chloroplast sequence data

The cpDNA *trn*T*‐trn*F sequence alignment had 1716 bp, and totally 13 cpDNA haplotypes (chlorotypes) were identified. Of these chlorotypes, 12 (H1–H12) were found in *H. gyantsensis*, one haplotype (H13) was obtained in *H. rhamnoides* subsp. *yunnanensis*. Chlorotype composition of each population was listed in Table 1.

All phylogenetic analyses for chlorotypes produced similar topology. The ML tree is shown in Figure 1a, in which chlorotypes from *H. gyantsensis* were divided between two clades (i.e. Clade A and B) with high support values. Molecular clock hypothesis could not be rejected by the likelihood ratio test (*p* = .75), and divergence times were calculated by the molecular clock model, shown in Figure 1a. The two lineages of *H. gyantsensis*, Clade A and Clade B, diverged at 3.6 Ma (95% highest posterior density, HPD: 2.2–5.0 Ma). The network of cpDNA haplotypes showed clades consistent with the phylogeny (Figure 1a). Chlorotype H9 from *H. gyantsensis*, located in the center of the star‐like structure, was most frequent and widely distributed. Strongly supporting the overall phylogeographic structure found in this study, the phylogenetic relationships based on all GenBank *trn*L‐*trn*F and ITS sequences also clearly demonstrated phylogenetic division between two geographic groups of populations. These groups closely corresponded to the main groups we identified (Appendix Figures S2 and S3).

Spatial distributions of all cpDNA haplotypes found in this study were shown in Figure 1b. Of the 12 chlorotypes of *H. gyantsensis*, four occurred only in one population respectively (private chlorotype, H4, H6, H11, and H12), while H9 was found in seven populations. Population P20 from eastern part of the range had the highest haplotype diversity (*H*_d_ = 0.754) and contained five of the 12 chlorotypes. The population of *H. rhamnoides* subsp. *yunnanensis* was monomorphic. Throughout the distribution of *H. gyantsensis*, chlorotypes in Clade A (yellow) were only found in the western part while those in Clade B (red and pink) only in the eastern part. Though population P6 and P11 containing chlorotypes of Clade A were spatially very close to P10 and P12 containing chlorotypes of Clade B, neither of them had chlorotypes from different clades (Figure 1b).

The populations of *H. gyantsensis* were divided into two groups according to SAMOVA, including the western group (P1–P9, P11) and the eastern group (P10, P12–P21), which was consistent with the phylogeny of *H. gyantsensis* Haplotype diversity (*H*_d_) was estimated to be 0.827, and the overall nucleotide diversity (π) was 1.68 × 10^−3^. In addition, total genetic diversity (*H*_T_) of *H. gyantsensis* was 0.865, and within population diversity *H*_S_ of each taxon was 0.151 (Table 2). *N*_ST_ (0.950) was significantly larger than *G*_ST_ (0.825) in the overall populations of *H. gyantsensis* (*p* < .001), indicating significant phylogeographic structure in this species, but such pattern was not detected separately within the western or the eastern group. The AMOVA result also revealed high genetic differentiation (*F*_st_ = 0.891), with 79.29% of genetic variation partitioned among groups, 17.68% among populations within groups, and only 3.03% within populations (Table 3).

**TABLE 2:** Results of genetic diversity, mismatch distribution analysis and neutrality tests in *H. gyantsensis* according to chloroplast *trn*T‐*trn*F data.

Groups	*H*_S_	*H*_T_	*G*_ST_	*N*_ST_	Mismatch analysis			Neutrality test	
					τ	SSD	HRI	Tajima's *D*	Fu's *F*s
*H. gyantsensis*	0.151	0.865	0.825	0.950*	27.750	0.065*	0.037*	2.470	33.520
*H. gyantsensis* western group	0.100	0.790	0.873	0.846	17.220	0.195*	0.238*	0.580	28.590
*H. gyantsensis* eastern group	0.198	0.655	0.698	0.730	1.020	0.004	0.064	−0.850	−1.760

Abbreviations: *G*_ST_ and *N*_ST_, genetic differentiation; HRI, Harpending's raggedness index; *H*_S_, mean genetic diversity within populations; *H*_T_, total genetic diversity; SSD, sum of squared deviation under expansion model.

* *p* < .05.

**TABLE 3:** Results for the analyses of molecular variance (AMOVA) for chloroplast and nuclear. microsatellite data of *H. gyantsensis.*

Source of variation	*df*	Sum of squares	Variance components	Percentage of variation	Fixation index (*F*_st_)
cpDNA trnT‐trnF					
Among groups	1	1709.785	11.531	79.29	
Among populations within groups	19	684.397	2.571	17.68	
Within populations	276	121.514	0.440	3.03	
Total	296	2515.697	14.542		0.891
Nuclear microsatellite					
Among groups	2	356.371	0.662	22.75	
Among populations within groups	18	378.187	0.532	18.28	
Within populations	743	1274.726	1.716	58.97	
Total	763	2009.284	2.910		0.410

Abbreviation: *df*, degree of freedom.

In all populations and the western group of *H. gyantsensis*, nucleotide mismatch distributions were multimodal, suggesting that the hypothesis of sudden expansion could not be accepted. However, mismatch distribution was unimodal in the eastern group of *H. gyantsensis* with *p*‐values of SSD and HRI larger than .05 (Table 2), which matched the sudden expansion model. Correspondingly, Tajima's *D* and Fu's *F*s showed negative values only in this group, but they were not significant (*p* > .05). The time of expansion in the eastern group estimated was at 0.12 Ma.

### 3.2 Microsatellite data

For the 11 microsatellite loci used here, genetic diversity indices were summarized for the population (Table 1) and each locus (Table 4). The number of different alleles per locus ranged from 1.524 to 5.571 with a mean of 2.545. Mean *H*_o_, *H*_e_, and PIC among loci were 0.212, 0.304, and 0.443, respectively (Table 4). Population P5 from west of central part of the range had the highest expected diversity (*H*_e_ = 0.437) and it simultaneously contained genetic components of three main lineages. Population P13 from east of central part of the range had the lowest diversity (*H*_e_ = 0.201, Table 1). Population differentiation ranged from 0.093 to 0.617 with the average value for multilocus estimates being 0.322. In addition, gene flow (*N*_m_) per locus varied from 0.155 to 2.425 with mean of 0.772.

**TABLE 4:** Genetic diversity, genetic differentiation, and gene flow of different microsatellite loci in *H. gyantsensis.*

Locus	*N*_a_	*I*	*H*_o_	*H*_e_	PIC	*F*_st_	*N*_m_
JSR01	1.524	0.165	0.096	0.096	0.109	0.139	1.551
JSR02	2.238	0.310	0.035	0.166	0.180	0.093	2.425
JSR03	5.571	1.300	0.267	0.627	0.898	0.309	0.559
JSR04	1.952	0.358	0.237	0.224	0.270	0.271	0.672
JSR05	2.190	0.425	0.088	0.247	0.605	0.617	0.155
JSR06	2.762	0.656	0.305	0.382	0.445	0.222	0.876
JSR07	2.000	0.466	0.351	0.307	0.478	0.439	0.320
JSR08	2.048	0.415	0.198	0.259	0.358	0.396	0.381
JSR09	2.571	0.618	0.449	0.382	0.614	0.436	0.324
JSR10	1.667	0.275	0.201	0.176	0.180	0.228	0.848
JSR11	3.476	0.847	0.107	0.473	0.741	0.395	0.383
Mean	2.545	0.530	0.212	0.304	0.443	0.322	0.772

Abbreviations: *F*_st_, genetic differentiation coefficient; *H*_e_, expected heterozygosity; *H*_o_, observed heterozygosity; *I*, Shannon's Information index; *N*_a_, number of alleles per locus; *N*_m_, gene flow; PIC, polymorphic information content.

The structure analysis based on microsatellite data suggested the optimal number of genetic groups was *K* = 3 (Figure 2a). Our gene flow analyses showed that both historical and contemporary levels of migration among the three genetic clusters were similar, but the historical migration values revealed little to no migration in contrast to contemporary migration rates (Figure 2b). To compare with chloroplast genetic structure, we also showed the result of *K* = 2, which was largely consistent with the distribution of two chloroplast and ITS clusters (Appendix Figures S2 and S3). The largest differences between the analyses based on chloroplast and microsatellite data were found in distribution of five populations (P7–P11). For chloroplast clusters, these populations except for P10 were possessed by the western cluster. However, for microsatellite data, they were mixed populations between western (*N*_1_) and eastern cluster (*N*_2_). When we chose *K* = 3 (the optimal number), five populations (P8–P10, P12–P13) located in central part of the range formed a new cluster (*N*_3_), and where two other populations 7 and 11 still showed mixed type. In addition, populations 5 and 6 possessed some genetic component of the new cluster (Figure 2c). When *K* = 4, the eastern cluster was divided into two genetic units (Figure 2c).The AMOVA result showed 22.75% of the variation was partitioned among groups, 18.28% among populations within groups, and the rest 58.97% within populations (Table 3). The differences of AMOVA result between microsatellite and chloroplast data were mainly resulted from their different evolutionary rate and history.

The ABC results suggested that a scenario that *N*_3_ derived from historical admixture between *N*_1_ and *N*_2_ (Scenario 4) was the most probable model with 30.07% posterior probability (Figure 3). According to the simulated results, there was no significant changes in effective population size between ancestral population and current populations. Posterior parameter estimates for Scenario 4 indicated that *N*_1_ and *N*_2_ diverged from each other at 65.5 ka (95% HPD: 17.5–132.0 ka), followed by a genetic admixture about 26.9 ka (Table 5).

**TABLE 5:** Posterior estimates of demographic parameters for the best model of population divergences based on Approximate Bayesian Computation.

Cluster	Parameter	Mean	Median	Mode	95% HPD
3	*N*_a_	1.55 × 10^4^	1.57 × 10^4^	1.94 × 10^4^	0.20–2.82 × 10^4^
	*N*_1_	1.04 × 10^4^	1.01 × 10^4^	0.88 × 10^4^	0.39–1.79 × 10^4^
	*N*_2_	1.14 × 10^4^	1.14 × 10^4^	0.11 × 10^4^	0.49–1.82 × 10^4^
	*N*_3_	1.00 × 10^4^	0.96 × 10^4^	0.84 × 10^4^	0.31–1.80 × 10^4^
	*t*_2_ (generations)	1.31 × 10^4^	1.19 × 10^4^	0.90 × 10^4^	0.35–2.64 × 10^4^
	*t*_2_ (years)	6.55 × 10^4^	5.95 × 10^4^	4.50 × 10^4^	1.75–13.2 × 10^4^
	*t*_1_ (generations)	5.39 × 10^3^	4.58 × 10^3^	3.40 × 10^3^	1.11–12.7 × 10^3^
	*t*_1_ (years)	2.69 × 10^4^	2.29× 10^4^	1.70 × 10^4^	0.55–6.35 × 10^4^
	*μ*	2.92 × 10^−5^	2.53 × 10^−5^	2.06 × 10^−5^	1.00–6.10 × 10^−5^
	*r*_a_	4.70 × 10^−1^	4.59 × 10^−1^	4.58 × 10^−1^	0.83–8.93 × 10^−1^

Abbreviations: *N*_a_, ancestral population size; *N*_1_, *N*_2_, and *N*_3_ represent effective population size of western cluster, eastern cluster, and central cluster, respectively. *t*, divergence time or admixture time of different clusters; *μ*, the mutation rate; *r*_a_, admixture rate.

### 3.3 Climate heterogeneity

**FIGURE 4:**
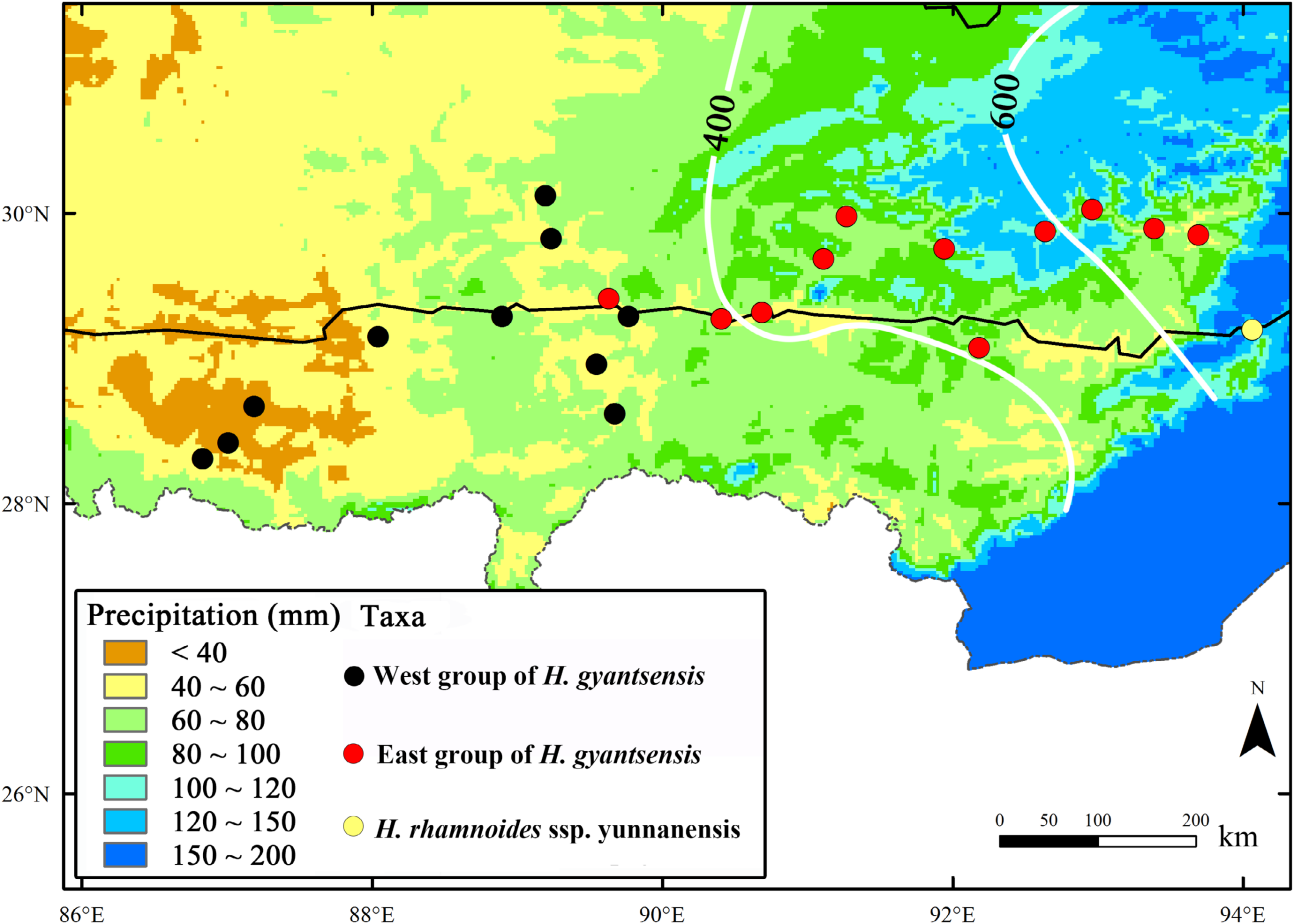
A map representing June precipitation in the EHHM region, based on the 30‐year average precipitation from 1961 to 1990 from the Climate Source (http://www.climatesource.com/), with the resolution of 1.25 arc‐minutes (~2 km). The white lines correspond to the 400 and 600 mm annual precipitation lines are adapted from Li et al. (2007).

**FIGURE 5:**
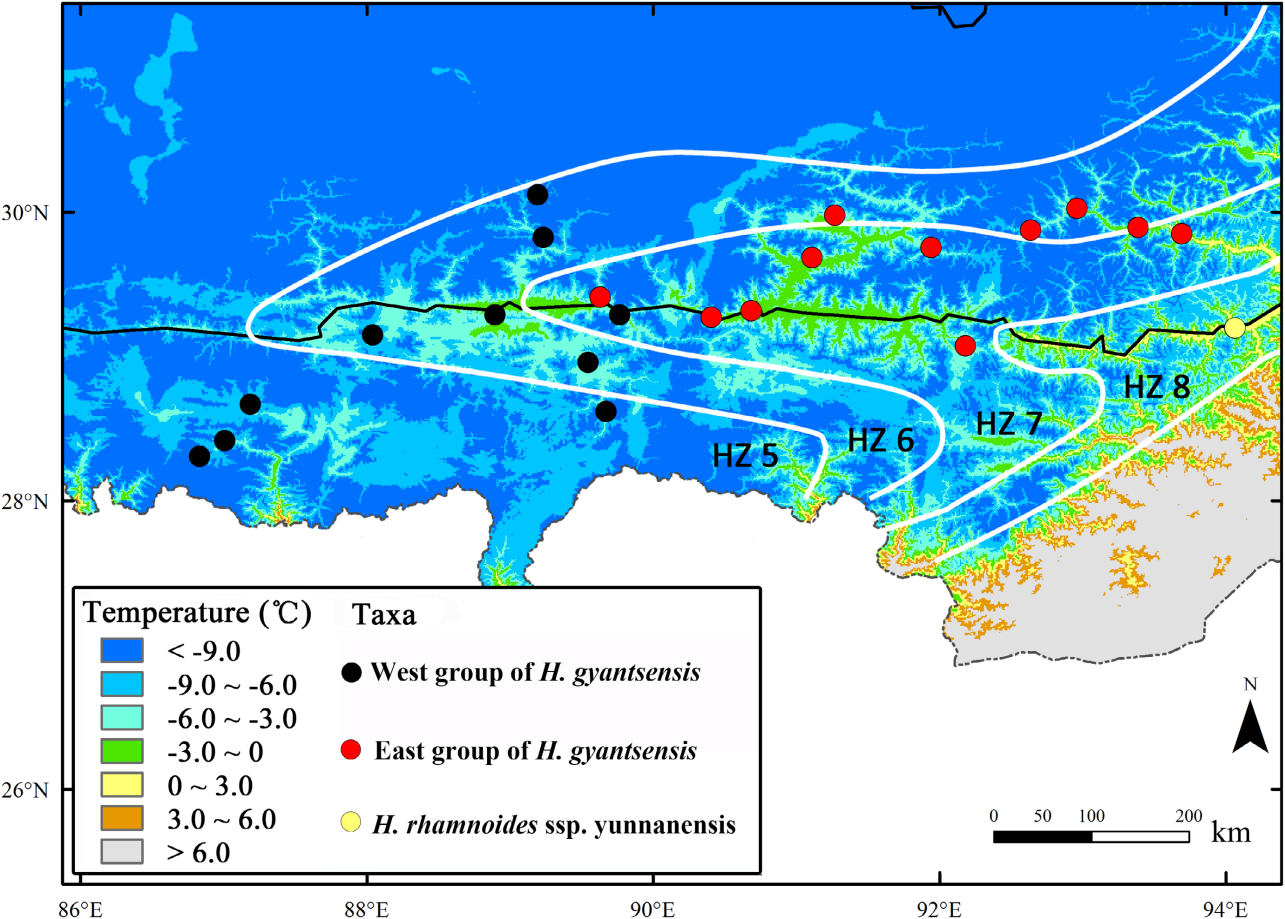
A map representing winter temperature in the EHHM region based on the BIOCLIM variable bio11 (mean temperature of coldest quarter); the white lines show plant hardiness zones in this region (HZ5–HZ8) according to China Plant Hardiness Zones published by Widrlechner (1997).

A total of 19 BIOCLIM variables were estimated in 21 populations and results were shown in Table 6. Out of the eight precipitation variables, annual precipitation (bio12), and precipitation seasonality (bio15) were significantly different between the two groups of *H. gyantsensis*. All the temperature variables (bio1–bio11) except isothermality (bio3) were also significantly different between these two groups (Table 6). After Bonferroni correction, annual mean temperature (bio1), minimum temperature of coldest month (bio6) and precipitation seasonality (bio15) were significantly different between them. The western group was limited to areas with an average of less than 60 mm of June precipitation while the eastern group to areas of 60–100 mm (Figure 4). Almost all populations of the eastern group were located in China Plant Hardness Zone (CPHZ) 7 (−17.7 to −12.3°C) while those of the western group in CPHZ 5 and CPHZ 6 (Figure 5).

**TABLE 6:** BIOCLIM variables in *H. gyantsensis* populations and the results of two‐tailed *t*‐tests between groups and analysis of variance (ANOVA).

	bio1 (°C)	bio2 (°C)	bio3 −1	bio4 (SD × 100)	bio5 (°C)	bio6 (°C)	bio7 (°C)	bio8 (°C)	bio9 (°C)	bio10 (°C)	bio11 (°C)	bio12 (mm)	bio13 (mm)	bio14 (mm)	bio15 (mm)	bio16 (CV)	bio17 (mm)	bio18 (mm)	bio19 (mm)
*H. gyantsensis* western group																			
P01	0.5	15.3	46	6186	15.0	−17.6	32.6	7.7	−6.3	8.3	−7.5	351	117	1	128	263	8	257	11
P02	3.0	15.6	47	6275	17.7	−15.3	33.0	10.3	−4.0	10.9	−5.1	421	137	1	127	313	7	310	11
P03	2.4	16.6	45	6923	18.6	−17.7	36.3	10.5	−5.4	11.1	−6.5	293	110	1	143	234	5	231	6
P04	3.8	15.7	44	6773	19.5	−15.6	35.1	11.4	−4.2	12.2	−5.1	349	123	1	137	274	3	272	3
P05	6.5	15.7	44	6644	22.1	−13.0	35.1	14.5	−2.4	14.5	−2.4	423	146	0	140	336	0	336	0
P06	5.0	15.0	44	6476	19.7	−14.0	33.7	12.9	−3.6	12.9	−3.6	315	98	0	132	242	0	242	0
P07	3.4	14.7	44	6396	17.4	−15.5	32.9	11.2	−5.1	11.2	−5.1	292	88	0	126	217	1	217	1
P08	1.8	14.4	41	7018	17.3	−17.0	34.3	9.8	−7.3	10.5	−7.3	289	98	0	137	230	0	227	0
P09	4.0	14.9	42	6885	19.6	−15.1	34.7	12.4	−5.1	12.4	−5.1	326	112	0	140	260	0	260	0
P11	6.1	14.9	44	6547	21.0	−12.7	33.7	14.1	−2.6	14.1	−2.6	369	113	0	133	282	0	282	0
Mean	3.7	15.3	44	6612	18.8	−15.4	34.1	11.5	−4.6	11.8	−5.0	343	114	0.4	134	265	2.4	263	3.2
SE	0.6	0.2	0.5	90	0.6	0.6	0.4	0.7	0.5	0.6	0.6	16	6	0.2	1.9	12	1.0	12	1.4
*H. gyantsensis* eastern group																			
P10	5.8	15.0	44	6611	20.9	−13.1	34.0	13.8	−3.0	13.8	−3.0	359	114	0	137	279	0	279	0
P12	7.4	14.6	44	6354	21.9	−10.9	32.8	15.1	−1.1	15.1	−1.1	429	124	0	126	320	2	320	2
P13	7.9	14.6	44	6298	22.4	−10.4	32.8	15.5	−0.6	15.5	−0.6	445	127	0	123	331	2	331	2
P14	7.9	14.6	44	6343	22.7	−10.2	32.9	15.6	−0.6	15.6	−0.6	441	132	0	129	335	2	335	2
P15	5.9	14.7	43	6636	20.9	−12.8	33.7	14.0	−2.2	14.0	−2.9	390	115	0	127	294	2	294	2
P16	5.1	15.0	44	6582	20.0	−13.8	33.8	13.2	−3.6	13.2	−3.6	347	99	0	125	255	1	255	1
P17	6.1	15.3	45	6324	20.5	−12.8	33.3	13.8	−2.3	13.8	−2.3	318	92	0	121	230	1	230	1
P18	3.7	14.9	44	6622	18.2	−15.3	33.5	11.8	−5.1	11.8	−5.1	349	93	0	113	243	2	243	2
P19	6.1	14.5	44	6471	20.5	−12.3	32.8	14.0	−2.6	14.0	−2.6	451	111	1	107	297	4	297	4
P20	7.9	13.9	44	6192	21.7	−9.5	31.2	15.4	−0.4	15.4	−0.4	538	123	1	101	337	5	337	5
P21	8.6	13.5	44	5981	22.1	−8.2	30.3	15.9	0.5	15.9	0.5	589	128	2	96	357	7	357	7
Mean	6.6	14.6	44	6401	21.1	−11.8	32.8	14.4	−1.9	14.4	−2.0	423	114	0.4	119	298	2.5	298	2.5
SE	0.4	0.2	0.1	62	0.4	0.6	0.3	0.4	0.5	0.4	0.5	25	4	0.2	3.8	13	0.6	13	0.6
*p* (*t*‐test)	.001*	.05	n.s.	.05	.05	.001*	.01	.01	.01	.01	.01	.05	n.s.	n.s.	.001*	n.s.	n.s.	n.s.	n.s.
Intergroup variance	0.94	0.88	–	0.79	0.91	0.95	0.87	0.94	0.94	0.93	0.94	0.87	–	–	0.93	–	–	–	–

Abbreviation: n.s.: not significant.

* Significant after Bonferroni correction.

## 4 DISCUSSION

### 4.1 Genetic diversity and phylogeographic structure

We found a relatively high level of total genetic diversity of chloroplast genomes (*H*_T_ = 0.87) in our sample. A very similar level of total genetic diversity was reported by Jia et al. (2016) for their sample of two chloroplast loci (*H*_T_ = 0.83). We also found that within population diversity of chloroplast genomes was two times higher in eastern than in western group (*H*_S_ = 0.20 and *H*_S_ = 0.10, respectively, Table 2). The considerably higher diversity of chloroplast genomes in the eastern group indicates that ancestral area of the species could be associated with this part of its current range. However, estimates from microsatellites suggested that the mean expected heterozygosity within populations was on average higher for populations from the western, than for populations from the eastern cluster (mean per group *H*_e_ = 0.34 and *H*_e_ = 0.28, respectively). The estimates of genetic diversity within populations based on SSR loci in our sample were generally at the lower range of similar estimates reported for other sets of microsatellite loci and other species of *Hippophaë* (Bartish and Thakur, 2022). These results may indicate lower level of genetic diversity in nuclear genome of *H. gyantsensis*, than in some other taxa from the genus, which have been sampled for similar analyses (*H. rhamnoides* subsp. *mongolica*, subsp. *sinensis* and subsp. *turkestanica*). The difference in genetic diversity may result from the considerably narrower range of *H. gyantsensis*, than ranges of the other three taxa.

Phylogeographic analysis of the cpDNA data showed that *H. gyantsensis* had a strong phylogeographic structure, and all sampled populations were divided into two distinct lineages, which occupy the western and eastern part of its range, respectively (Figure 1b). In the revealed phylogeny, all the chlorotypes from the western group represented a strongly supported clade while all the chlorotypes from the eastern group formed another strongly supported clade, and the mean age of divergence between the two lineages was estimated at ~3.6 Ma (Figure 1a). This estimate is within the range (1.2–3.9 Ma) reported by Jia and Bartish (2018) for the crown node of the species. Therefore, both age estimates (the earlier study and ours) placed the earliest diversification in *H. gyantsensis* confidently into the Pliocene/Pleistocene epochs. Finally, we should note that Jia et al. (2016), using a different sample of 17 populations of *H. gyantsensis* from approximately the same area and a combined data set of two chloroplast loci (*trn*L‐*trn*F and *trn*S‐*trn*G) and ITS fragment of nuclear DNA, did not find any phylogeographic structure in the species. The result was based on non‐significant difference between *G*_ST_ and *N*_ST_ in their data. These authors explained the lack of phylogeographic structure in the species by a complex history of colonization by gene pools that had been genetically distinct for a long period and spread across the whole distribution range. However, we note that reported by Jia et al. (2016) maps of chloro‐ and ribotypes suggest existence of two distinct geographically‐defined clusters of populations, which closely correspond to the eastern and western groups in our study. Besides, these authors failed to include in their sample the most eastern part of the range of *H. gyantsensis* (the area is represented by populations P18–P21 in our study, Appendix Figure S4). This omission could result in an unbalanced representation of populations from the two groups reported in our study in the sample of Jia et al. (2016). Consequently, the omission could result in lack of significance in the analysis of difference between *G*_ST_ and *N*_ST_ reported by Jia et al. (2016) for their sample of populations. This interpretation is further supported by higher values of *G*_ST_ and *N*_ST_ in our sample (0.83 and 0.95, respectively), than in the earlier study (0.70 and 0.63, respectively). Besides, our phylogenetic analyses of all the public *trn*L‐*trn*F and ITS sequences also confirmed the existence of two main groups of populations in *H. gyantsensis* (Appendix Figures S2 and S3).

We found no phylogeographic structure within the two main groups of populations. Compared with *H. tibetana*, *H. gyantsensis* has a narrower and continuous distribution and most populations of this species grow along the valleys of the YZR (Lian et al., 2006). In addition, fruits of *Hippophaë* plants constitute important food sources of many bird species (Lu et al., 2005) and can therefore be dispersed over relatively long distances. These facts strongly suggest that there are no obvious strong geographic or biotically defined barriers between most populations of this species, especially between the populations along the boundary of the two groups. In fact, the boundary populations (P6, P10, P11, and P12) are very close to each other geographically and are linked by confluent rivers, so that seeds of their individual plants could be dispersed across this boundary easily by birds. However, the genetic split between two lineages is very clear in space. In addition, our gene flow analyses showed that historical levels of migration among the three genetic clusters revealed little to no migration, further excluding the historic gene flow. These results, a long temporal divergence between the lineages, a short spatial distance, and obvious opportunities for long‐distance dispersal of seeds between the ranges of two lineages, indicate that geographic barriers are unlikely to drive the formation of phylogeographic structure of this species and the divergence of the two lineages.

### 4.2 Effects of climate factors on the formation of phylogeographic structure of *H. gyantsensis*

Previous studies have found significant phylogeographic structure in plant species from the EHHM region (Ge et al., 2005; Li et al., 2011; Qiu et al., 2011). The formation of such structure is often attributed to vicariance, as gene flow is often blocked by geographical barriers (e.g. high mountains). However, some many studies also found that the split between lineages does not strictly follow the geographic barriers, but geological isolation and ecological factors, especially climate, together promote the divergence of several species from this region (Fan et al., 2013; Liu et al., 2013; Yang et al., 2012; Zhang et al., 2020). In the study on *H. tibetana*, the split between lineages coincided with 400 and 600 mm annual precipitation lines, also suggesting that climate played an important role in driving intraspecific diversification in *H. tibetana* (Wang et al., 2010). Here, a pattern similar to *H. tibetana* was found in *H. gyantsensis*, including the similar dividing line.

Local adaptation, resulting from natural selection of different ecological factors, may play an important role in the formation of phylogeographic structures in addition to geographic isolation (Avise, 2000; Fournier‐Level et al., 2011). Though the YZR spreads across a limited range of latitude (28°~30°), many ecological factors in this region vary greatly from west to east, including climate, vegetation, and soil (Liu, 2010; The Editorial Committee of Vegetation Map of China CAOS, 2007). Between the above two groups, all the temperature variables (bio1–bio11) of BIOCLIM except isothermality (bio3) showed significant differences in average values (Table 6). Among these 10 temperature variables, minimum temperature of coldest month (bio6) had the largest *F*‐value (17.98) and the highest variance between two groups (0.95), suggesting that minimum temperature of the coldest month may be a key factor to drive the split of *H. gyantsensis* into two groups of populations with significantly different climatic adaptations. In the EHHM region, the minimum temperature of coldest month is equivalent to annual minimum temperature (The Comprehensive Scientific Expedition to the Qinghai‐Xizang Plateau, 1984). The latter identifies the location of environmental conditions under which a species or variety of plant can successfully survive and grow (McKenney et al., 2007) and is the basis of defining plant hardiness zones (Widrlechner, 1997). Figure 5 shows plant hardiness zones in the EHHM according to China Plant Hardiness Zones (CPHZ) published by Widrlechner (1997). It was found that almost all populations of the eastern group were located in CPHZ 7 (−17.7 to −12.3°C) while those of the western group in CPHZ 5 or 6.

In the QTP, precipitation has also been considered as a key climate factor to determine the divergence of species, and annual precipitation lines of 400 and 600 mm were two important boundaries for different lineages of *H. tibetana* (Wang et al., 2010). Between the western and eastern parts of *H. gyantsensis*, precipitation seasonality (bio15) have also showed significant differences in average values (Table 6), and their boundary was very near the annual precipitation line of 400 mm (Figure 4). In this study, the relevance of genetic divergence of *H. gyantsensis* with June precipitation was also analyzed due to its potential importance and for obtaining more accurate data. As the beginning of the India monsoon every year, June precipitation is not only very closely related to the precipitation seasonality on the QTP, but is also an important factor for the growth and development of *H. gyantsensis* since this plant is flowering in this month (Crimmins et al., 2011). Figure 4 shows the spatial gradient of June precipitation in the EHHM region and indicates its impact on the distribution of *H. gyantsensis*. As shown in the figure, the western group was limited to areas with an average of less than 60 mm of June precipitation, while the eastern group to areas of 60–100 mm.

Another two important ecological factors, vegetation and soil, did not have significant differences between eastern and western ranges of *H. gyantsensis*; instead, four populations of the east group (P18–P21) grow in a different vegetation and soil zone (The Editorial Committee of Vegetation Map of China CAOS, 2007), suggesting low impact of these two ecological factors on phylogeographic structure in *H. gyantsensis*. These results show that the heterogeneity of climate in the reaches of YZR is the key factor to limit the dispersal of *H. gyantsensis* and to drive their lineage divergence.

### 4.3 Recent regional uplift of the QTP and effects on the formation of phylogeographic structure of *H*. *gyantsensis*

As discussed before, the climate heterogeneity in the reaches of YZR is the most important and direct factor to drive the divergence and phylogeographic structure of *H. gyantsensis*. This heterogeneity is mainly determined by two key factors: the HM and Indian monsoon (IM). The climatic heterogeneity in the YZR occurred only when the HM reached a particular elevation and IM was driven up to certain strength. Therefore, the origin of the above heterogeneous climate must be accounted for timing of the rise of the HM, and the establishment of the IM.

Although both the time and course of their uplifts have been controversial (Deng & Ding, 2015), most of available so far evidences supported the suggestion that QTP and the HM have different histories (Deng & Ding, 2015; Li et al., 2015; Spicer et al., 2021; Su et al., 2019; Wang et al., 2014). The QTP might had risen to the recent height as early as 40 Ma (Tada et al., 2016; Wang et al., 2014; Wang, Schluetz, & Liu, 2008; Wang, Zhao, et al., 2008), but the uplift of the HM was s likely later than that of the QTP (Deng & Ding, 2015; Ding et al., 2017). Some studies on the basis of oxygen isotope indicate that the HM had reached its modern height or even higher than present by 20 Ma (Ding et al., 2017; Gébelin et al., 2013). However, some other studies suggested that before ~10 Ma, the HM were still dissected by some of the deepest and most impressive gorges on Earth and rivers rising in Gangdese (Transhimalaya) would have flowed across the HM and flowed south into Indian plain (Tremblay et al., 2015). In addition, fossils in Gyirong and Zhada showed these regions (the north and center of the HM) were still warm and moist in the late Miocene and even in the early Pliocene (Huang et al., 2020; Huntington et al., 2015; Ji et al., 1980), strongly suggesting the HM was unlikely to reach the recent height before the late Miocene. Furthermore, the massive conglomerate which has long been regarded as the product of orogeny around the QTP margin began to deposit at about 3.6 Ma, implying the central HM underwent a rapid rise in the Pliocene (Li et al., 2015). All these studies showed that different evidence from different regions of the QTP may reflect discrepant evolution history. This interpretation makes sense if: (1) the QTP is not a single geological entity but a fusion of several accreted terranes, and they evolve in piece‐meal manner (Fielding, 1996; Spicer et al., 2021; Su et al., 2019; Tapponnier et al., 2001; Wang, Schluetz, & Liu, 2008; Wang, Zhao, et al., 2008); (2) different types of data measure different aspects of the topography. For example, fossils are inclined to reflect low elevations, while isotopes tend to reflect high altitude (Botsyun et al., 2016; Spicer et al., 2021). Studies in phylogeography (microevolution) and phylogeny (macroevolution) frequently relate divergence‐time estimates to paleogeographic or climate events, often infer the abiotic factors making for clade diversification or speciation (Favre et al., 2015; Renner, 2016). Most phylogeographic, phylogenetic and biogeographic studies on taxa of the QTP were linked to rapid and recent uplift of the QTP (the late Miocene and later; e.g., Cheng et al., 2017; Li et al., 2013; Meng et al., 2017; Wang et al., 2005, 2010). Renner (2016) indicated the possible dilemma of the above linking to the conclusion of the QTP has been 4–5 km high since the mid‐Eocene through integrating different types of evidences, and argued that numerous biogeographic studies in which recent uplift of the QTP caused the species differentiation just were self‐created bubble. However, the differentiated uplift of the QTP and HM has been confirmed by so many studies, of which the elevation history of the QTP should not be lumped together with the history of recent uplift of HM. Indeed, some regions of the QTP reached a high elevation in the QTP since the mid‐Eocene, but other regions, especially the HM, have a younger uplift history (Deng et al., 2011; Wang et al., 2012; Zhang et al., 2012; Zheng et al., 2000). These results indicated that the possibility of species differentiation, especially those with current ranges at high altitudes and adapted to alpine ecosystems, was affected by the younger geological events.

In addition, the IM is another key factor to determine the climate of the EHHM, which has been considered to be closely related to the uplift of the HM and QTP (Boos & Kuang, 2010; Tada et al., 2016). Regarding the onset of IM, more recent studies suggested that this appears to have begun during the late Middle Miocene (~12.9 Ma; Betzler et al., 2016) or even earlier (Guo et al., 2002; Huber & Goldner, 2012) and summer monsoon was in its full strength in the late Miocene (~7 Ma). Despite some controversy in understanding of the relationship between IM development and HM uplift, the perception that IM intensification occurred in the Late Pliocene is rarely disputed (An et al., 2001; Lu et al., 2020; Zhang & Liu, 2010; Zheng et al., 2004). By this time, the divergence of the two lineages of *H. gyantsensis* may had occurred, according to our estimates. This case adds therefore support to the hypothesis of partially recent and rapid QTP uplift. The concordances between geological, climatic, and biotic processes imply that the likely recent uplift of HM acted as creator of a high climatic heterogeneity in this region. The reinforcement of Indian monsoon can also be associated with the divergence of this species. Moreover, some species divergences could be better explained by even more recent geological uplift (Wang et al., 2010; Xing & Ree, 2017). The possible concordance of age of divergence between the two main lineages of *H. gyantsensis* revealed in this study and the currently accepted age of Qingzang Movement (Li et al., 2015), further indicated strong links between geo‐climatic and biotic processes in the region.

### 4.4 Demographic history and population genetic admixture

In this study, we simultaneously used chloroplast and microsatellite data to estimate the demographic history of *H. gyantsensis*. Theoretically, these two types of data can respectively explain ancient and more recent evolutionary history due to the differences in mutation rate (Bai & Zhang, 2014). Besides, nuclear and chloroplast genomes are transmitted between generations in different ways. Only nuclear genome information is carried by pollen, while both nuclear and chloroplast genomes are dispersed via seeds in *Hippophaë* (Bartish et al., 2002), as in most other angiosperms. The difference in transmission routes from parent to offspring generations for different genomes (chloroplast and nuclear) can lead to differences in effective population sizes between exclusively maternally (via seeds) transmitted chloroplast genome, and both maternally and paternally (via pollen) transmitted nuclear genome. For strictly outcrossing dioecious *Hippophaë*, this means, in theory, that (all else being equal) effective population sizes measured from sequences of chloroplast genes are one‐quarter of correspondent values based on nuclear genes (Birky et al., 1983).

For chloroplast data, we found east–west phylogeographic break in this species, and identified population expansion in the eastern cluster at about 0.12 Ma, when the TP was in the last interglacial period (MIS 5, Cui et al., 2011; Zheng et al., 2002). Similarly, a strong population genetic structure was also estimated from microsatellite data, but with three clusters (i.e. eastern cluster, central cluster, and western cluster). However, these three clusters have not displayed signs of demographic expansions in recent time (Table 5). Furthermore, ABC modeling indicated that the origin of central cluster (P8–P10, P11–P12) was a result of admixture between clusters eastern and western at 27 ka (Table 5). Interestingly, chloroplast haplotype network showed the two lineages were distant to each other (Figure 1), and both chloroplast and microsatellite data showed low gene flow (*N*_m_ = 0.03 for cpDNA; *N*_m_ = 0.772 for SSR). These results indicated that genetic admixture event was likely not a result from long distance dispersal in *H. gyantsensis*. Taken together, it is most likely that populations of eastern cluster have experienced demographic expansion during in the warm inter‐glaciation, and then met populations of western cluster around 27 ka, i.e., in an epoch which corresponds to the warm inter‐glaciation (MIS 3c) during the last glaciation (Cui et al., 2011). This westward dispersal event can be evidenced by the genetic composition of P7 and P11. These populations possessed chlorotypes of the western group, while microsatellite data revealed genetic components of both eastern and western clusters. The significant phylogeographic break in cpDNA data but not in nuclear data for most species was often explained by genetic introgression across spatially narrow admixture cline (Barton & Gale, 1993; Cheng et al., 2021; Li et al., 2019). The indicated that demographic expansion of eastern cluster of *H. gyantsensis* likely provided opportunity for the genetic introgression of mixed populations. Additionally, the time estimates of population expansion and genetic admixture event in this study were both traced back to warm inter‐glaciation during the Quaternary glaciation in China. Taken together, these results are potentially highlighting the importance of consequences of Quaternary climatic fluctuations for regional biota (i.e. glacial and interglacial isolation) to the recent evolutionary history of *H. gyantsensis* (Liu et al., 2014; Wen et al., 2014).

## 5 CONCLUSION

Our chloroplast and microsatellite analyses of *H. gyantsensis* clearly revealed that range of *H. gyantsensis* was most likely limited to the middle YZR in the Late Neogene. We also found significant phylogeographic structure in the species with strong spatial differentiation into eastern and western groups. Age of divergence between the two main lineages of the species could be traced to ~3.6 million years ago. A combination of the recent regional uplift of the QTP and HM and IM intensification could be among main factors triggering this event. Results of this study suggested that steep climatic gradients but not geographic barriers in the EHHM shaped phylogeographic structure of *H*. *gyantsensis*. The role of high mountains in the evolution of *H. gyantsensis* seems largely to be in creation of highly heterogeneous climates by affecting the flow of the Indian monsoon in this region. In addition, our results revealed a population expansion in the eastern cluster at about 0.12 Ma and a likely genetic admixture between the two main clusters at 27 ka, both events closely associated with the most recent interglacial and interstadial intervals. These findings suggested the recent regional orogeny, IM intensification, and Quaternary climatic fluctuations all had considerable impact on recent evolutionary history of *H. gyantsensis*.

## AUTHOR CONTRIBUTIONS

**Ting Xu:** Formal analysis (equal); writing – original draft (equal). **Ruixue Wang:** Investigation (equal); resources (equal). **La Qiong:** Investigation (equal); resources (equal). **Takahiro Yonezawa:** Methodology (equal); software (equal). **Xinyi Huang:** Methodology (equal); software (equal). **Sun Kun:** Investigation (equal); resources (equal). **Zhiping Song:** Validation (equal). **Yuguo Wang:** Methodology (equal); software (equal). **Igor V. Bartish:** Software (equal); writing – review and editing (equal). **Wenju Zhang:** Project administration (equal); supervision (equal); writing – original draft (equal). **Shanmei Cheng:** Formal analysis (equal); project administration (equal); validation (equal); writing – original draft (equal); writing – review and editing (equal).

## CONFLICT OF INTEREST STATEMENT

The authors declare no conflicts of interest.

## Supporting information


Appendix S1

## Data Availability

Sequences used in this study are deposited in NCBI's GenBank (accession nos. KJ542834–KJ542846, KJ542860).
