# Supplementary material for: Climate heterogeneity shapes phylogeographic pattern of Hippophae gyantsensis (Elaeagnaceae) in the east Himalaya‐Hengduan Mountains
Source: Ecol Evol. 2023 Jun 9;13(6):e10182. doi: 10.1002/ece3.10182 (PMC10251425; doi:10.1002/ece3.10182)
Supplement: Supplementary file 1 — Appendix S1 [file ECE3-13-e10182-s001.docx]

**APPENDIX**

| Locus | Primer (5'–3') | motif | *T*a(°C) | Size |
| --- | --- | --- | --- | --- |
| JSR01 | F-CAACCCAAATTTCACTTTGTCTC  R-GGACATTGTTGTCCTCTTCTCTG | (TTC)5 | 55 | 131-146 |
| JSR02 | F-ATAGAGCAAAAACCGGAAAGAAA  R-TGACAAAACGTCGAGGAAATTAT | (TCT)5 | 55 | 152-167 |
| JSR03 | F-TCCACCAAGAGGCTATGTAGTTC  R-AAGCGTCATCTCTGACAAAACTC | (AT)8 | 55 | 164-210 |
| JSR04 | F-GCATCTCCACTACCATGTCCTAC  R-AGAAGATGAGGACTTTGCAACTG | (CAT)5 | 55 | 162-177 |
| JSR05 | F-TTTTGAAACTTTTATTGAGAGTGC  R-TCTTTGTTCATTTGGCTATCTGG | (AT)7 | 55 | 159-169 |
| JSR06 | F-GATTTCAGAATCATTGGGTTCAC  R-ATGCTGTAGATCATTGCACTTCC | (GCT)5 | 55 | 155-167 |
| JSR07 | F-TTTTTATGCTTGTGGTAGTGGTG  R-AATTGATCACGAAAAGGCAATTA | (AT)6 | 55 | 148-152 |
| JSR08 | F-GGCGGTTTTCAAACTTATCTCTC  R-TTGACTTTTCCCAACAGTAGTGC | (TCC)5 | 55 | 160-169 |
| JSR09 | F-ACATTTGGGATCAGACTTTAGCC  R-GGCCTATTCAAGGGAGTAGTTTG | (AC)6 | 55 | 173-179 |
| JSR10 | F-CAATTGTTCAATACTAAATG  R-ATCCTAATCAAAAGAAATC | (A)6(CAAACA)3 | 50 | 130-136 |
| JSR11 | F-TGGATTAATGGAGAAAG | (AG)10 | 55 | 154-178 |
|  | R-CAAGGACAACAAAGACA |  |  |  |

**Table S1** The information on 11 microsatellite loci used in this study

**Table S2** Description of the prior distribution of parameters from eight scenarios in Figure 3 used in Approximate Bayesian Computation.

| Parameter | Distribution | Minimum | Maximum |
| --- | --- | --- | --- |
| Effective population size |  |  |  |
| *N*_1_ | Uniform | 10 | 20000 |
| *N*_2_ | Uniform | 10 | 20000 |
| *N_3_* | Uniform | 10 | 20000 |
| *N*_a_ | Uniform | 10 | 30000 |
| Time of events (in generations backward in time) |  |  |  |
| *t*_1_ | Uniform | 10 | 30000 |
| *t*_2_ | Uniform | 10 | 30000 |
| Admixture rate |  |  |  |
| *r_a_* | Uniform | 0.001 | 0.999 |
| Mean mutation rate |  |  |  |
| *μ* | Uniform | 1 × 10^-6^ | 1 × 10^-3^ |

*N*_1_, *N*_2_, and *N*_3_, current population sizes; *N*_a_, ancestral population size;. *t*_2_ and *t*_1_, divergence time or admixture time of different clusters, and *t*_2_ > *t*_1_.


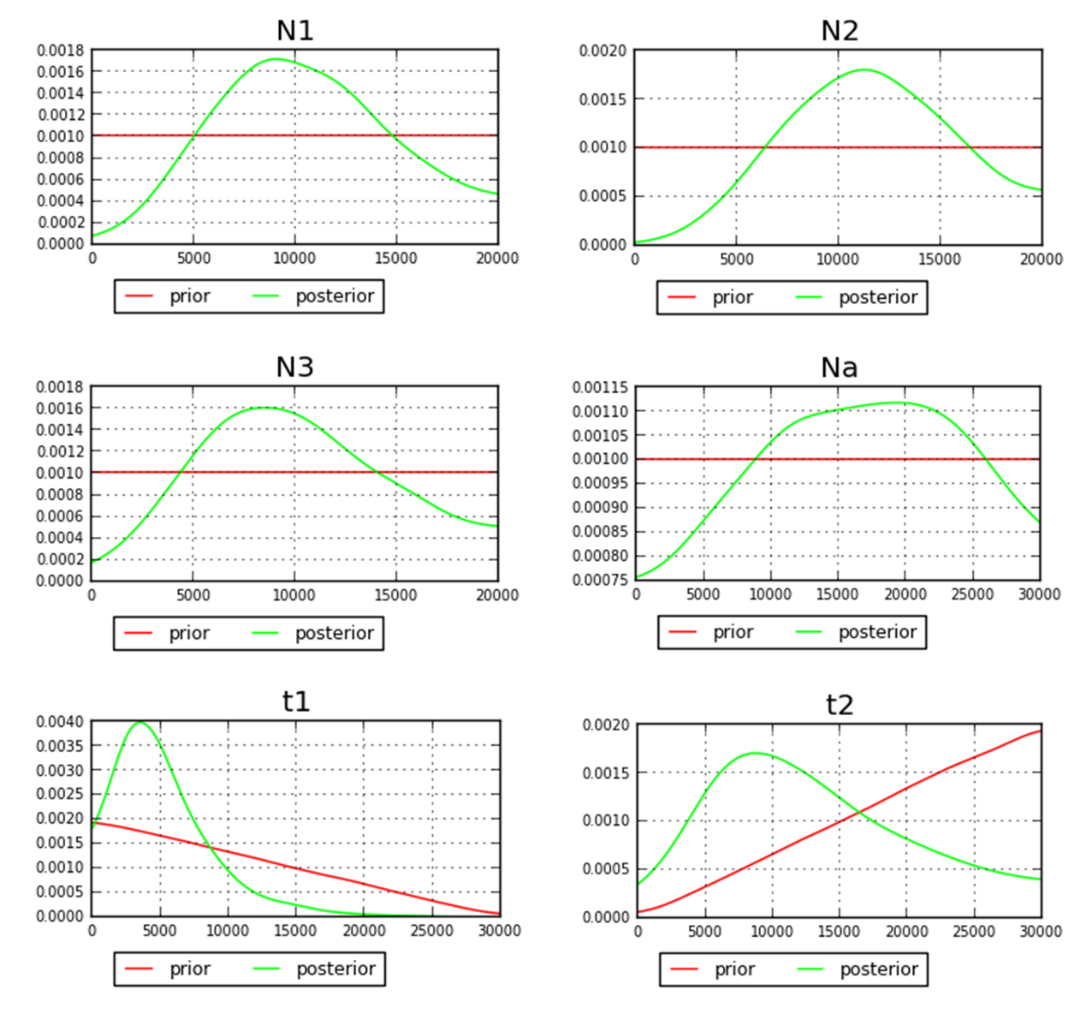


**Figure S1** Prior and posterior distributions of demographic parameters under Scenario 4 in figure 3 estimated by DIYABC


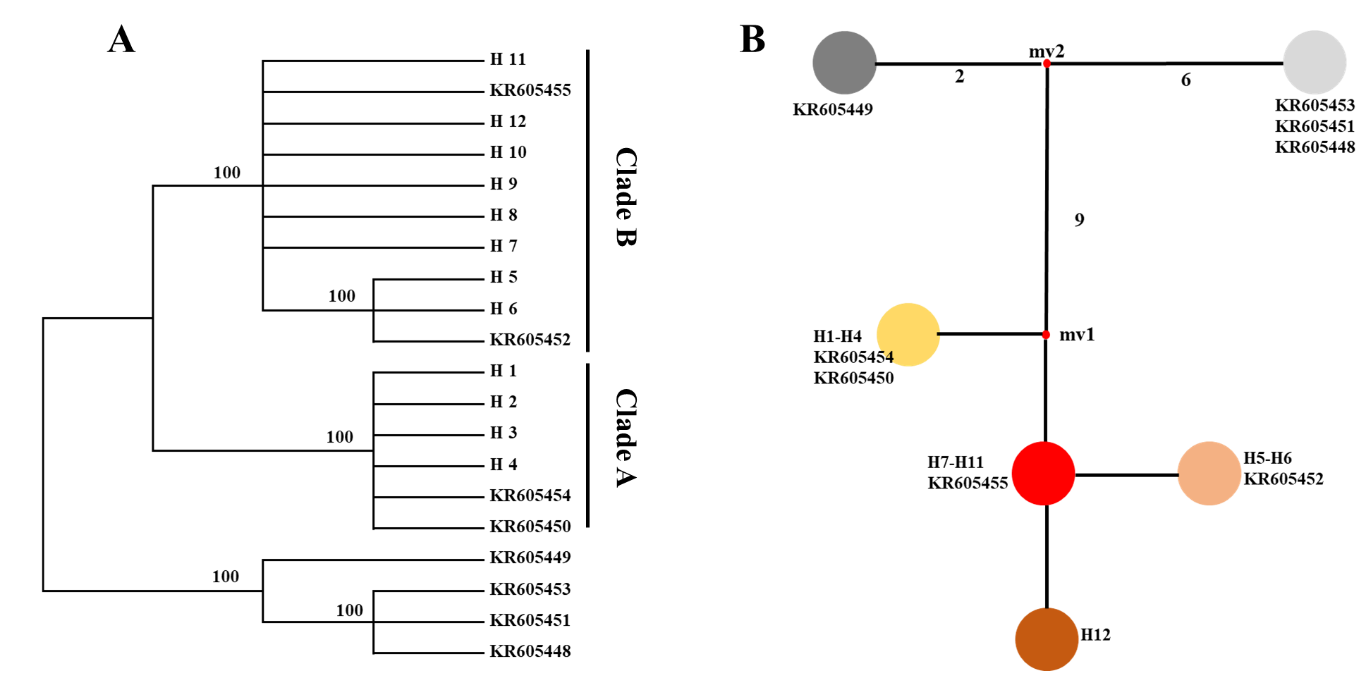


**Figure S2** Phylogenetic tree (A) based on ML method and median-joining network (B) of the *trn*L-*trn*F haplotypes of *H. gyantsensis* from Jia et al. (2016) and our study. Numbers near the branches are bootstrap values (A) and mutational steps (B), respectively. The other numbers in (B) represent single mutational steps.


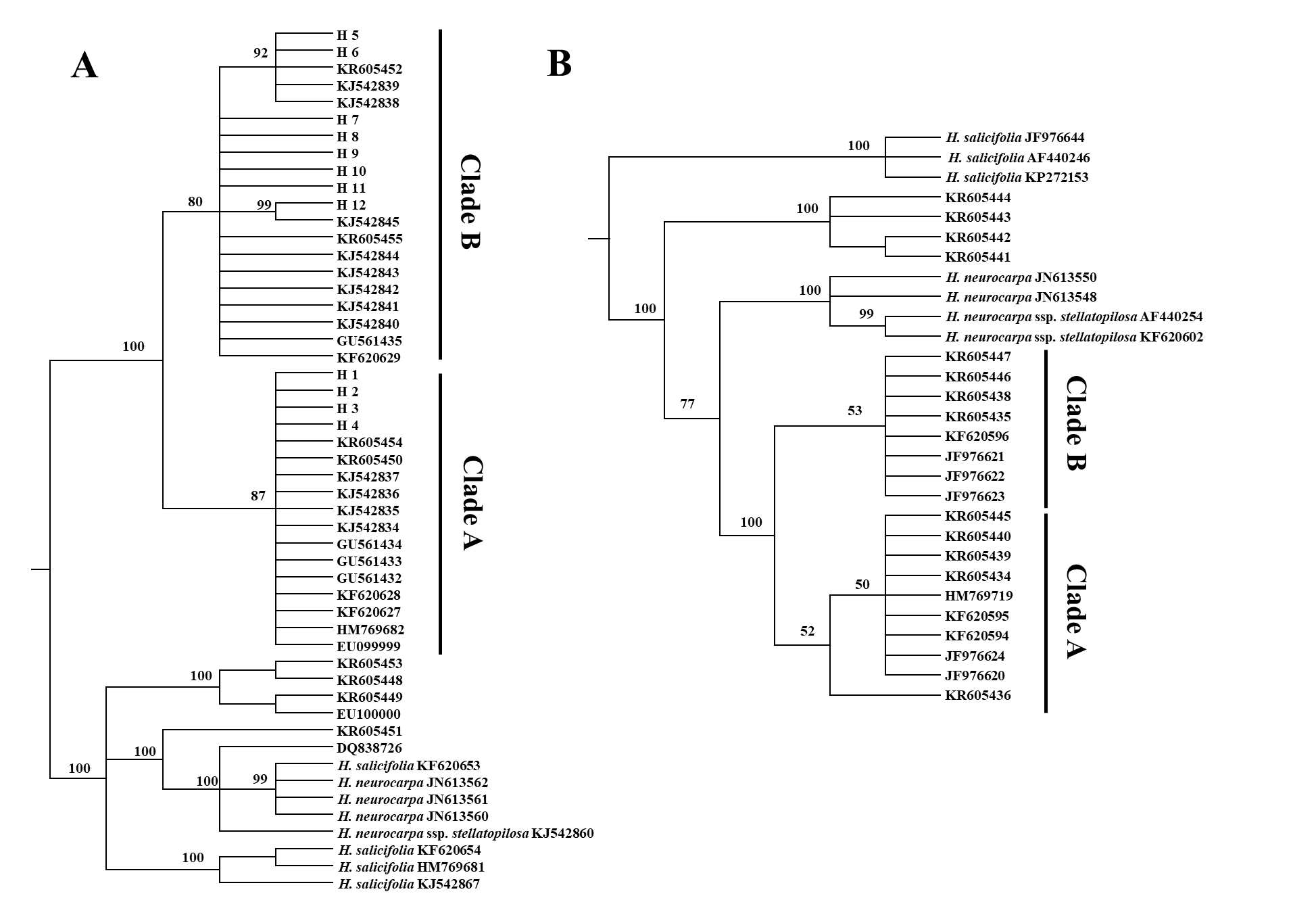


**Figure S3** Phylogenetic trees of all the public *trn*L-*trn*F (A) and ITS (B) sequences of *H. gyantsensis* downloaded from GenBank based on Bayesian analysis. Numbers above the branches are Bayesian posterior probabilities.


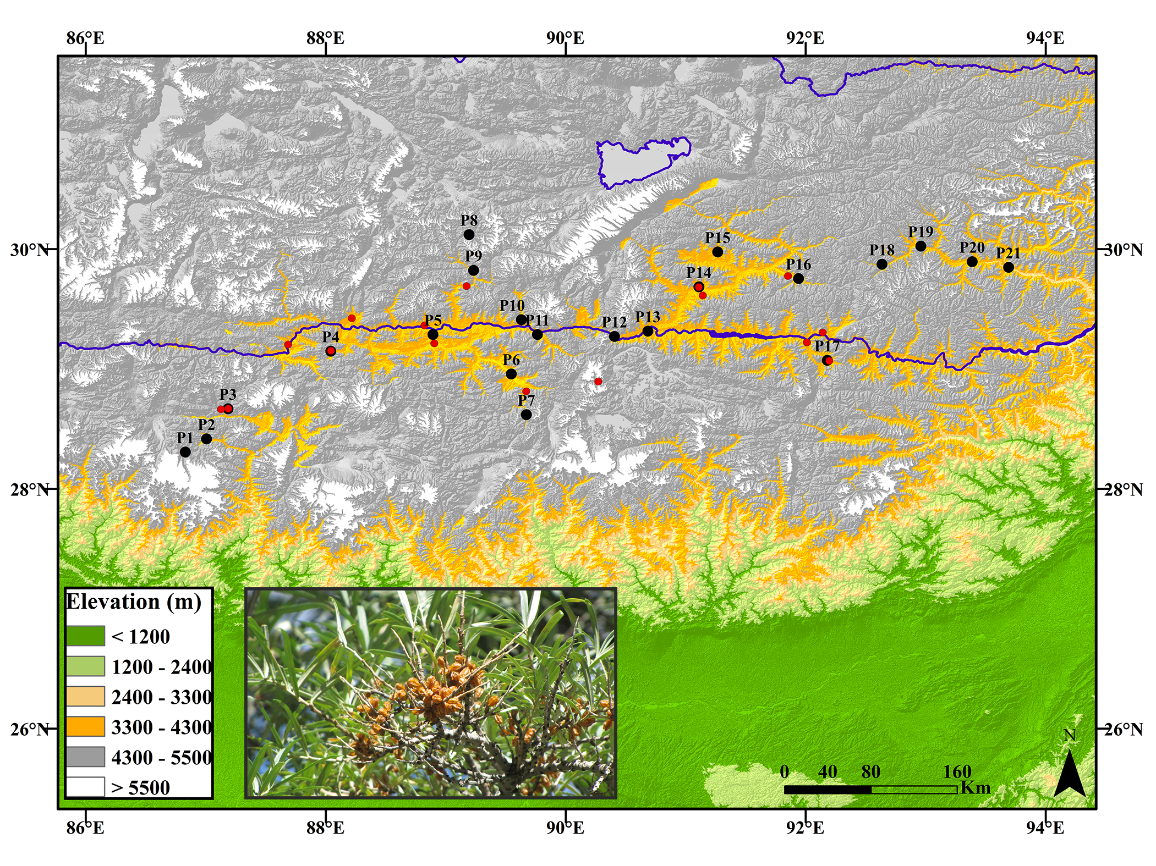


**Figure S4** The geographic distribution of localities of sampling of *H. gyantsensis* from this study (black circles) and Jia et al. (2016, red circles).
